# Tannic acid label indicates abnormal cell development coinciding with regeneration of renal tubules

**DOI:** 10.1186/1472-6890-14-34

**Published:** 2014-07-15

**Authors:** Will W Minuth, Lucia Denk

**Affiliations:** 1 Department of Molecular and Cellular Anatomy, University of Regensburg, University Street 31, D-93053 Regensburg, Germany

## Abstract

**Background:**

Stem/progenitor cells are in the focus of research as a future therapeutic option to stimulate regeneration in diseased renal parenchyma. However, current data indicate that successful seeding of implanted stem/progenitor cells is prevented by harmful interstitial fluid and altered extracellular matrix. To find out possible parameters for cell adaptation, the present investigation was performed.

**Methods:**

Renal stem/progenitor cells were mounted in an artificial interstitium for perfusion culture. Exposure to chemically defined but CO_2_-independent culture media was tested during 13 days. Cell biological features were then analyzed by histochemistry, while structural details were investigated by transmission electron microscopy after conventional and improved fixation of specimens.

**Results:**

Culture of renal stem/progenitor cells as well in Leibovitz’s L-15 Medium as CO_2_ Independent Medium shows in fluorescence microscopy spatial development of numerous tubules. Specimens of both media fixed by conventional glutaraldehyde exhibit in electron microscopy a homogeneous cell population in developed tubules. In contrast, fixation by glutaraldehyde including tannic acid illuminates that dispersed dark marked cells of unknown function are present. The screening further demonstrates that the dark cell type does not comply with cells found in embryonic, maturing or matured renal parenchyma.

**Conclusions:**

The actual data show that development of abnormal cell features must be taken into account, when regeneration of renal tubules is simulated under in vitro conditions.

## Background

Numerous papers published during the last years illustrate that an implantation of stem/progenitor cells appears as an innovative therapeutic alternative to treat acute and chronic renal failure [1,2]. However, critical reading of related literature also elucidates that this approach still moves more in a phase of basic research than in sound clinical trials. One of the obstacles is the minimal survival of implanted stem/progenitor cells limiting in turn successful regeneration of parenchyma [3].

Further on, implantation of stem/progenitor cells for regeneration of diseased renal parenchyma is not done with a simple injection but is only one of the links in an unexpected complex biomedical process. Literature informs, for example, that stem/progenitor cells can be principally administered via the arterial or venous vessel system, by punctual injections into diseased parenchyma or by seeding in the space left between the organ capsule and the outer parenchyma [4,5]. The various results illustrate that irrespective of applied implantation technique the expectations have not been achieved yet.

One has further to consider that before and during implantation stem/progenitor cells naturally occur within a special niche environment or are kept in the beneficial atmosphere of an individual culture medium [6,7]. However, when an implantation is performed, the environment for stem/progenitor cells drastically changes. Exposure to degenerating epithelia, altering extracellular matrix, unbalanced electrolytes, growth factors, interleukins and hormones supports inflammation but does not promote development of implanted cells [8-11]. The small fraction of surviving stem/progenitor cells has to migrate then to the molecular site of necessary restoration for turning the harmful environment into an atmosphere pushing repair of parenchyma. But how can it be realized, when stimulating interstitial fluid and attractive extracellular matrix are lacking. In this situation it appears that from stem/progenitor cells is required more than they can really perform.

To investigate developmental capacity in relation to environmental stress under in vitro conditions, renal stem/progenitor cells can be mounted in a pad consisting of a polyester fleece [5,12,13]. In this scenario the fibers of the fleece mimic extracellular matrix, while the space between acts as a reservoir for interstitial fluid. For controlled culture the artificial interstitium is then placed in a perfusion container, where contained cells are provided with always fresh nutrition and respiratory gas by a constant transport of medium.

In the present set of experiments renal stem/progenitor cells were exposed to media containing different buffer systems stabilized against atmospheric air. Influence on developmental capacity was then recorded by cell biological methods and transmission electron microscopy. The actual data demonstrate for the first time that regenerated tubules contain beside normal also abnormal epithelial cells. Comparisons show that described abnormal cells are not contained in embryonic, maturing or matured renal parenchyma.

## Methods

### Preparation of renal stem/progenitor cells

Care, use of animals and performed experiments are in accordance with the Animal Ethics Committee, University of Regensburg, Regensburg, Germany. Kidneys from one-day old New Zealand rabbits (Seidl, Oberndorf, Germany) were isolated under sterile conditions and cut into a ventral and dorsal half as earlier described [14]. Then the fibrous organ capsule was stripped off by fine forceps to obtain a constantly thin layer of stem/progenitor cell niches adherent to the explant. Applying this method embryonic tissue of up to 1 cm in square can be isolated.

### Offering an artificial interstitium

**Figure 1:**
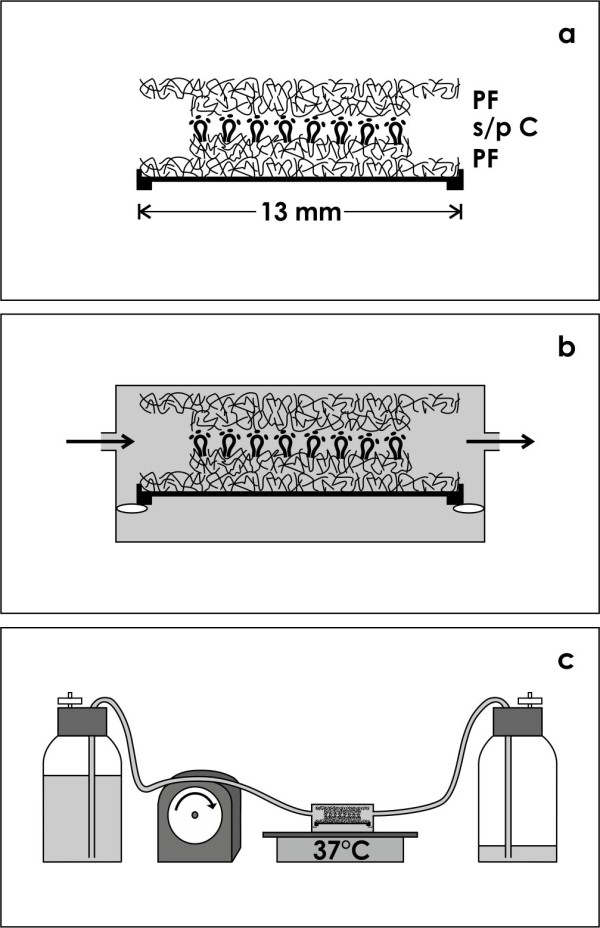
**Schematic illustration shows generation of tubules by renal stem/progenitor cells. a)** After isolation renal stem/progenitor cells (s/pC) are placed between layers of polyester fleece (PF) to create an artificial interstitium. The fleece layers are mounted in a Minusheet® tissue carrier. **b)** For culture the carrier is transferred to a perfusion container with horizontal flow characteristics. **c)** Perfusion culture is performed for 13 days under atmospheric air. During this period always fresh culture medium is transported by a peristaltic pump (1.25 ml/h) from a storage bottle to the container and then to a waste bottle.

To analyze development of renal tubules an isolated embryonic tissue layer containing numerous stem/progenitor niches was mounted in an artificial interstitium as it was earlier described [5,13]. Briefly, the isolated tissue was placed between punched out layers (5 and 13 mm in diameter) of polyester fleece (I7, Walraf, Grevenbroich, Germany) and mounted inside a Minusheet® tissue carrier (Minucells and Minutissue, Bad Abbach, Germany) (Figure 1a). The tissue carrier was transferred to a perfusion culture container with horizontal flow characteristics (Figure 1b). Then for a period of 13 days always fresh culture medium was continuously transported at a rate of 1.25 ml/h with an IPC N8 peristaltic pump (Ismatec, Wertheim, Germany) (Figure 1c). To work independently from a CO_2_-incubator, all of the culture experiments were performed under atmospheric air on a thermo plate (Medax-Nagel, Kiel, Germany) at 37°C.

### Chemical defined culture media

For the present experiments chemical defined Leibovitz’s L-15 Medium (Nr. 31415–029, 34 cultured specimens) and CO_2_ Independent Medium (Nr. 18045–054, 43 cultured specimens) all including Phenolred (GIBCO/Invitrogen, Karlsruhe, Germany) were applied. Infections were prevented by adding an antibiotic-antimycotic cocktail (1%, GIBCO/Invitrogen). Development of tubules was induced by application of aldosterone (1 × 10^−7^ M, Fluka, Taufkirchen, Germany) as it was earlier described [5]. The pH of 7.4 under atmospheric air is stable in CO_2_ Independent Medium. To obtain this pH in Leibovitz’s L-15 Medium, N-2-Hydroxyethylpiperazine-N-2-Ethane Sulfonic Acid (HEPES; GIBCO/Invitrogen) was added by titration.

### Histochemistry on generated tubules

After run of a perfusion culture experiment the polyester fleeces 13 mm in diameter with adherent renal tubules were fixed in 70% ethanol for whole mount label. Before labeling the specimens were washed several times with phosphate buffered saline (PBS, pH 7.5) and incubated for 30 minutes with blocking solution (PBS, 10% horse serum from GIBCO/Invitrogen, 1% bovine serum albumin from Serva, Heidelberg, Germany). Then the undiluted antibody anti-cytokeratin 19 (TROMA III, Developmental Studies Hybridoma Bank DSHB, Iowa, USA) was applied for one hour. After washing with 1% BSA in PBS the specimens were incubated for 45 minutes with goat-anti-rat-IgG-rhodamine (Jackson Immunoresearch Laboratories, West Grove, USA) diluted 1:50 in PBS containing 1% BSA. Following several washes with PBS the labeled specimens were analyzed using a CM12 confocal laser scanning microscope (Zeiss, Oberkochen, Germany). Fluorescence images were taken with a digital camera at a standard exposure time and thereafter processed with Corel DRAW Graphic Suite X5 (Corel Corporation, Otawa, Canada). In parallel, screening for renal cell features was performed with fluorescent lectins such as BPL (Bauhinia Purpurea Lectin), GSL (Griffonia Simplicifolia Lectin), LTL (Lotus Tetragonolobus Lectin), WGA (Wheat Germ Agglutinin), DBA (Dolichos Biflorus Agglutinin), PNA (Peanut Agglutinin) and SBA (Soybean Agglutinin) all from Vector (Burlingame, USA).

### Transmission electron microscopy (TEM)

The polyester fleeces 5 mm in diameter containing generated tubules were harvested after 13 days of culture and cut in four parts. Kidneys were surgically removed and then prepared. To obtain a correct orientation of parenchyme along the cortico-medullary axis of lining collecting ducts, tissue blocks between both organ poles were specifically excised as it was performed earlier [15].

In the present investigation specimens were analyzed after conventional fixation in glutaraldehyde (GA) and after improved contrasting by GA solution including tannic acid as it was earlier described [14,16].

For fixation the following solutions were used:

1. Specimens for control: 5% GA (Serva) buffered with 0.15 M sodium cacodylate, pH 7.4.

2. Specimens for improved contrast with tannic acid: 5% GA buffered with 0.15 M sodium cacodylate, pH 7.4 + 1% tannic acid (Sigma-Aldrich Chemie, München, Germany).

Primary fixation was performed for 1 day at room temperature. After several washes with 0.15 M sodium cacodylate the samples were postfixed in the same buffer but additionally containing 1% osmium tetroxide (Science Services, München, Germany). Then specimens were washed with sodium cacodylate buffer and dehydrated in graded series of ethanols.

Finally, specimens were embedded in Epon (Fluka, Taufkirchen, Germany), which was polymerized at 60°C for 48 h. Semithin and ultrathin sections were made with a diamond knife on an ultramicrotome EM UC6 (Leica, Wetzlar, Germany).

Semithin sections of specimens fixed by glutaraldehyde were stained with Richardson solution, while specimens fixed by glutaraldehyde including tannic acid were analyzed without further staining. Analysis of semithin sections was performed with an Axioscope microscope (Zeiss). Images were taken with a digital camera (AxioCam MRC, Zeiss) and thereafter processed with Corel DRAW Graphic Suite X5 (Corel Corporation).

Ultrathin sections were collected onto slot grids coated with 1.5% Pioloform (Plano, Wetzlar, Germany) and contrasted using 1% uranyl acetate and lead citrate as it was earlier described [17]. Analysis of ultrathin sections was performed at 80 kV using an EM 902 transmission electron microscope (Zeiss).

### Definition of cells within the renal stem/progenitor cell niche

For the presented experiments embryonic parenchyma derived from the outer cortex of neonatal rabbit kidney was analyzed containing renal stem/progenitor cell niches. The nomenclature of previously published papers was used [18].

## Results

During the process of implantation stem/progenitor cells are transferred from a beneficial culture medium to the inflammatory environment of diseased parenchyma. To obtain first basic information, if such a harsh transition can be balanced to a certain degree, renal stem/progenitor cells were embedded in a polyester fleece as a substitute for interstitial extracellular matrix and exposed to chemically defined media. In the present series the influence of CO_2_-stabilized culture media on tubule development was tested.

### Fluorescence microscopy on generated tubules

**Figure 2:**
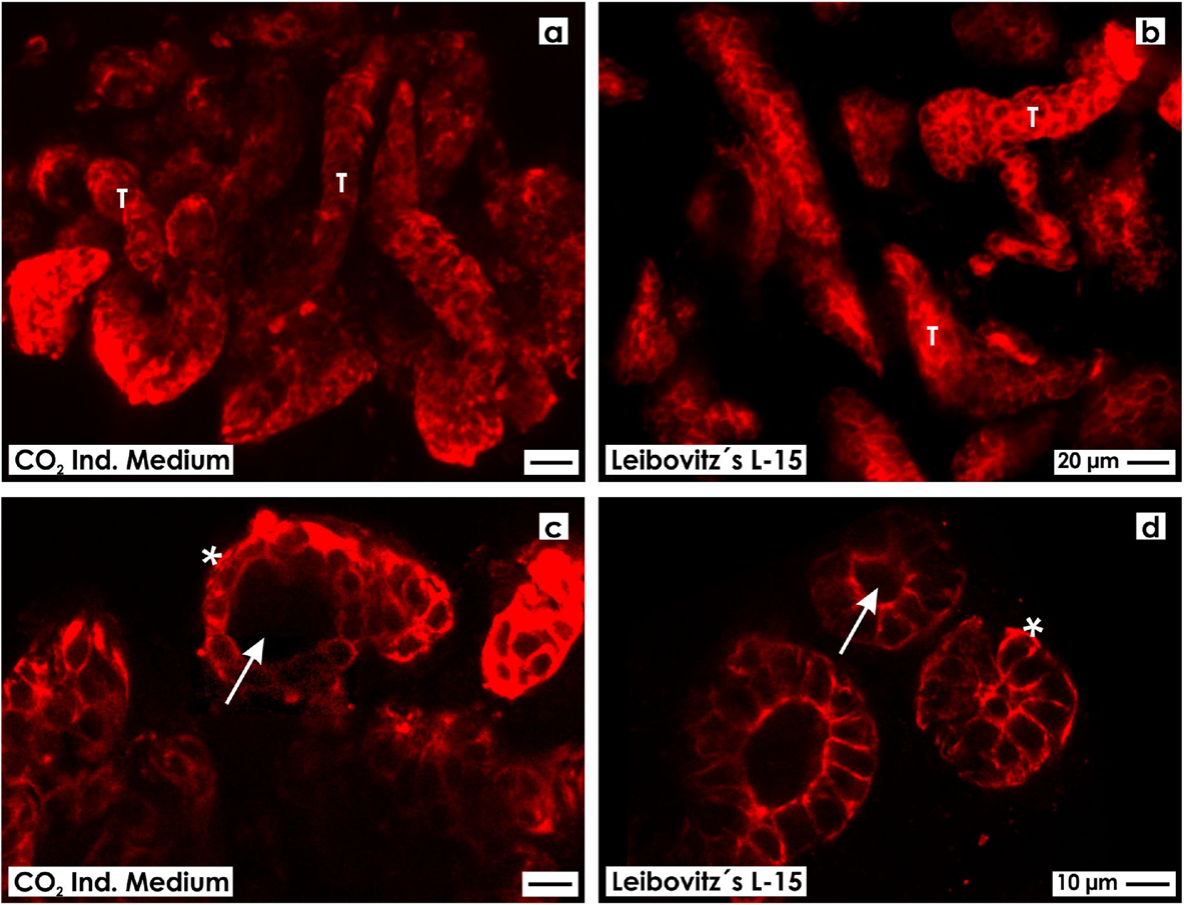
**Confocal fluorescence microscopy on whole mount species labeled for cytokeratin 19 (Troma III) to analyze spatial distribution of generated tubules.** As well in **(a)** CO_2_ Independent Medium and **(b)** Leibovitz’s L-15 Medium development of numerous tubules (T) is observed within the artificial interstitium. **c,d)** Higher magnification depicts that label is detected on all of analyzed cells. Further generated tubules contain a simple epithelium with prismatic cells bordering a lumen (arrow) and a basal lamina (asterisk).

After 13 days of perfusion culture whole mount label was performed on harvested specimens to analyze the degree of spatial development of regenerated tubules (Figure 2). As well in series with CO_2_ Independent Medium (Figure 2a) as in series with Leibovitz’s L-15 Medium (Figure 2b) confocal fluorescence microscopy points out that numerous tubules labeled by TROMA III are developing between the polyester fibers of the artificial interstitium. Further can be seen that generated tubules of both CO_2_ Independent Medium (Figure 2c) and Leibovitz’s L-15 Medium (Figure 2d) show an epithelium with prismatic cells. In selected cross and longitudinal sections a lumen and a continuously developed basal lamina is visible.

Fluorescence microscopy also elucidates that SBA label illustrates numerous collecting duct tubules developing between the polyester fibers of the artificial interstitium. This marker indicates that most probably collecting duct tubules are contained as it was earlier described (no figure) [5,13]. To recognize features typical for proximal tubule cells binding of further lectins was tested. Astonishingly, only a barely visible reaction on single tubules was found in series labeled by BPL, while no label was detected in series with GSL, LTL and WGA. In none of the cases label on single cells within the tubule epithelium was registered (no figure).

### Light microscopical analysis

**Figure 3:**
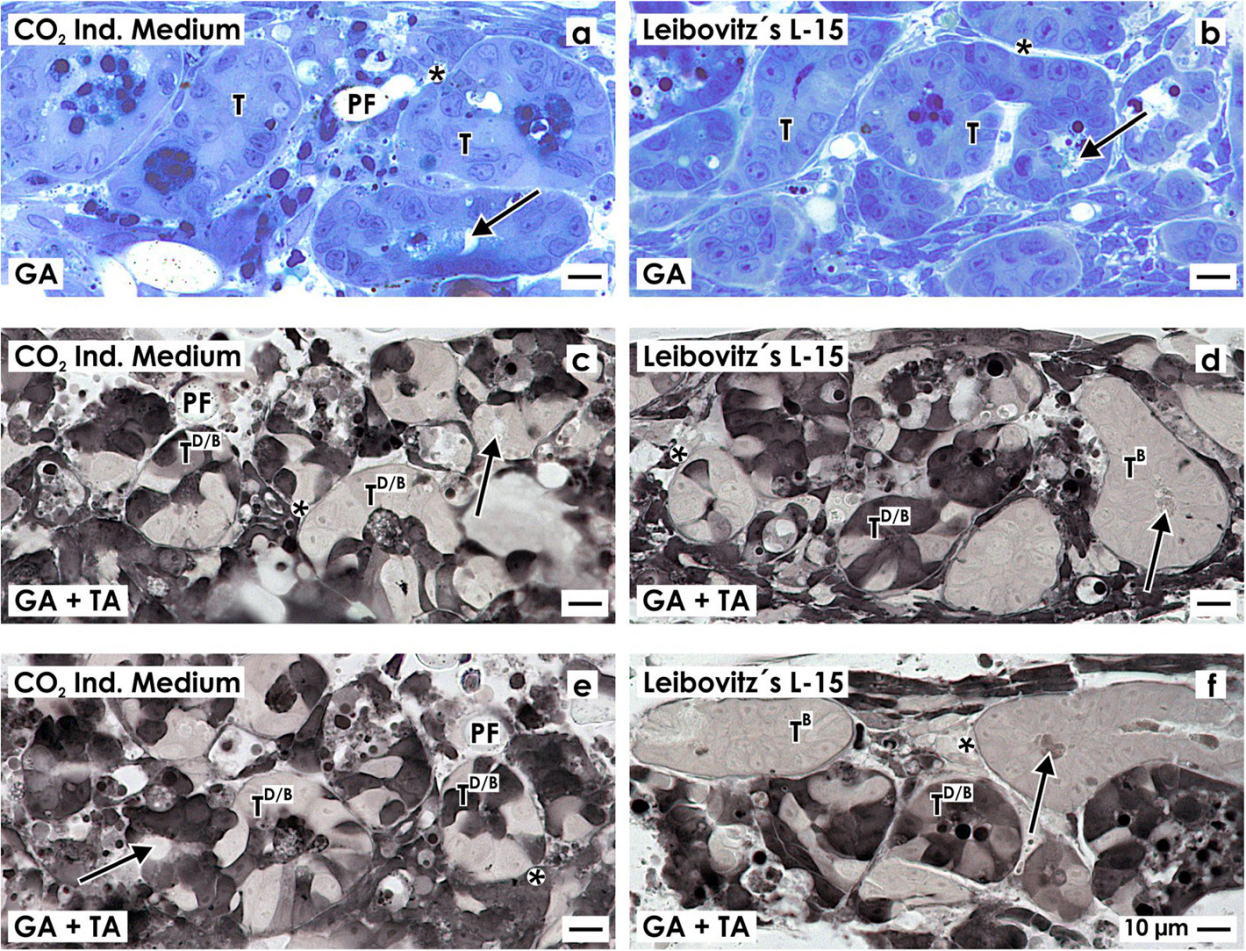
**Light microscopy on semithin sections demonstrates numerous tubules (T) generated between fibers of the polyester fleece (PF).** Development of tubules is observed in series with **(a,c,e)** CO_2_ Independent Medium and **(b,d,f)** Leibovitz’s L-15 Medium. Generated tubules exhibit a lumen (arrow) and a basal lamina (asterisk). **a,b)** Fixation of specimens in conventional glutaraldehyde followed staining by Richardson solution shows tubules with an inconspicuously looking homogeneous cell population. In contrast, specimens generated in **(c,e)** CO_2_ Independent Medium or **(d,f)** Leibovitz’s L-15 Medium but fixed by glutaraldehyde containing tannic acid show tubules with a dark and bright cell population (T^D/B^) or tubules containing only bright cells (T^B^).

In the following set of experiments semithin sections were produced to obtain more information about morphological details of tubule cells generated in CO_2_ Independent Medium (Figure 3a,c,e) and Leibovitz’s L-15 Medium (Figure 3b,d,f).

Specimens fixed in conventional glutaraldehyde show after staining with Richardson solution that numerous tubules are present within the artificial interstitium (Figure 3a,b). In series with either CO_2_ Independent Medium (Figure 3a) or Leibovitz’s L-15 Medium (Figure 3b) cross sections illustrate that a single layer of a homogeneous cell population with a basal lamina is bordering a lumen. Differences in morphological quality of cells cannot be recognized. In several cases the lumen is filled with apoptotic cells and a luminal matrix as it is known from other developing epithelia [19]. Vacuoles in the cytoplasm of generated tubule cells are rare. This result indicates that toxic effects or non appropriate chemical compounds are obviously not present in selected culture media.

In contrast, specimens fixed in glutaraldehyde containing tannic acid show after culture with CO_2_ Independent Medium (Figure 3c,e) and Leibovitz’s L-15 Medium (Figure 3d,f) a completely different cell pattern as compared to series fixed in conventional glutaraldehyde (Figure 3a,b). Fixation by glutaraldehyde including tannic acid reveals that two different phenotypes of tubules are contained within the artificial interstitium. While the one type of tubules shows a homogeneously composed bright cell population (T^B^), the second type exhibits a heterogeneously composed epithelium consisting of dark and bright cells (T^D/B^). Thus, fixation of specimens in glutaraldehyde including tannic acid demasks hidden details and illuminates a heterogeneously composed cell population in the epithelium of generated tubules (Figure 3c,e and d,f).

### TEM on generated tubules fixed by glutaraldehyde

**Figure 4:**
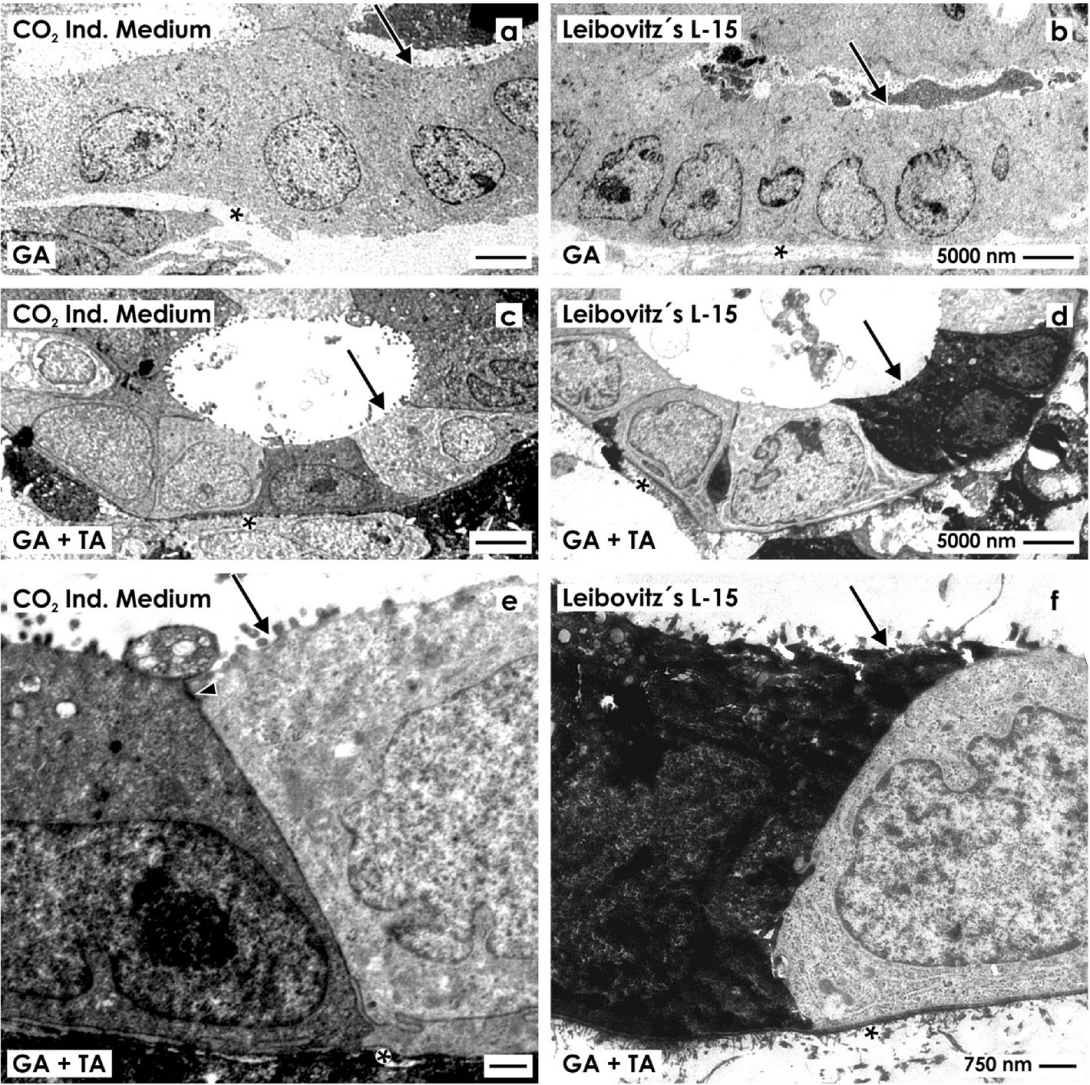
**Transmission electron microscopy of renal tubules generated in (a,c,e) CO**_**2 **_**Independent Medium and (b,d,f) Leibovitz’s L-15 Medium for 13 days.** Specimens fixed by **(a,b)** conventional glutaraldehyde show only bright tubule cells. In contrast, specimens fixed in **(c-f)** glutaraldehyde containing tannic acid reveal a heterogeneously composed epithelium consisting of bright and dark cells.** e,f)** High magnification shows intact bright and dark cells. The apical plasma membrane faces a lumen (arrow), while the basal side rests on a basal lamina (asterisk). Luminal and lateral plasma membranes are separated by a tight junction (arrow head).

To obtain more information about the heterogeneous cell population, transmission electron microscopy (TEM) was performed (Figure 4).

Specimens generated in CO_2_ Independent Medium (Figure 4a) or Leibovitz’s L-15 Medium (Figure 4b) fixed in conventional glutaraldehyde show tubules containing a polarized epithelium with intact morphology. In both cases the cytoplasm is looking inconspicuously. The luminal side of cell faces a lumen occasionally filled with apoptotic cells and some luminal matrix. The basal side of the epithelial cells rests on a continuously developed basal lamina.

In contrast, samples generated in CO_2_ Independent Medium (Figure 4c,e) or Leibovitz’s L-15 Medium (Figure 4d,f) fixed with glutaraldehyde including tannic acid show tubules including a polarized epithelium with intact morphology. Tubule segments were found, where bright cells dominate, while in other segments an equal number of bright and dark cells is visible. In both series the cytoplasm looks intact. In screened cases the luminal plasma membrane of cells is bordering a lumen. Finally, the basal plasma membrane is in contact with a continuously developed basal lamina consisting of a lamina rara, lamina densa and lamina fibroreticularis.

**Figure 5:**
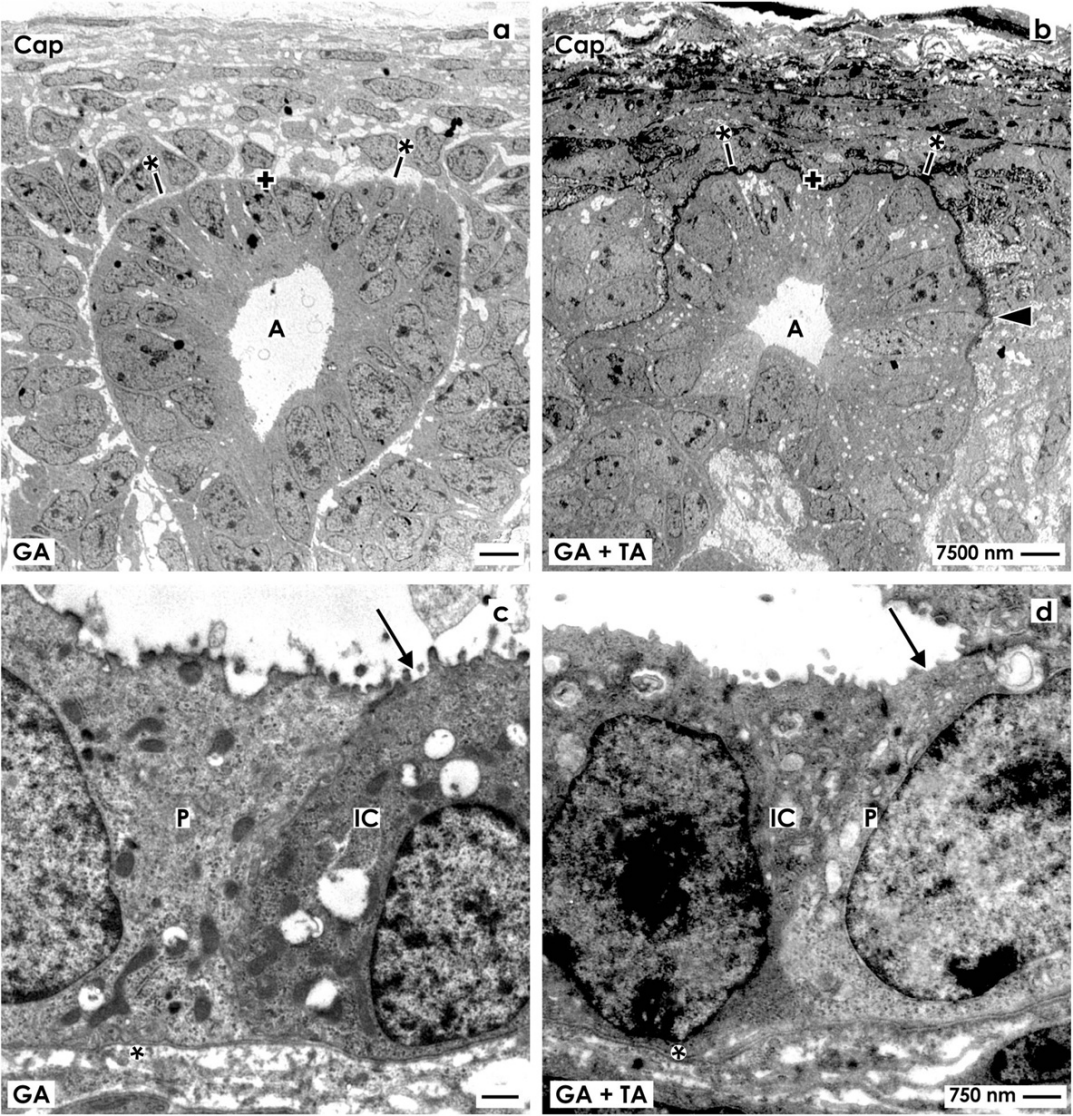
**Transmission electron microscopy of the stem/progenitor cell niche in neonatal rabbit kidney after fixation of specimens by (a,c) conventional glutaraldehyde and (b,d) glutaraldehyde including tannic acid. a)** Neither in a collecting duct ampulla (A) nor in surrounding mesenchymal stem/progenitor cells or organ capsule (Cap) a dark cell type is detected. The epithelial-mesenchymal interface is bright (asterisk with bar). The basal aspect of a CD ampulla is marked by a cross. **c)** At the ampulla neck maturation of light ‘Principal Cells’ and grey ‘Intercalated Cells’ is seen. **b)** Fixation of specimens in glutaraldehyde including tannic acid shows intense label at the epithelial-mesenchymal interface (asterisk with bar) as it was earlier described [15]. **d)** Label of tannic acid is lost at the lateral side of the ampulla (arrow head). Neither in embryonic, maturing or matured renal parenchyma dark cells within a tubule epithelium are present.

Higher magnifications of series with CO_2_ Independent Medium (Figure 4e) and Leibovitz’s L-15 Medium (Figure 4f) illustrate that between the luminal and lateral plasma membranes as well on bright as dark cells an intact tight junction complex is developed. This finding is a hint that a physiological sealing is established between neighboring cells. Further the intercellular space between bright and dark cells is not pathologically extended but appears to be narrow. In all of analyzed cases a close basal slit is found at the contact site between the basal and lateral plasma membranes. On the lateral plasma membranes only few interdigitating microvilli respectively folds are found.For control, to find out whether newly detected dark cells in generated tubules (Figure 4c-f) have similarities with a related cell population in renal parenchyma, a further screening by transmission electron microscopy was performed (Figure 5).Specimens fixed by conventional glutaraldehyde illustrate a stem/progenitor cell niche in the outer cortex of neonatal kidney. In the center the tip of an ureteric bud derived collecting duct (CD) ampulla can be recognized (Figure 5a). Contained epithelial stem/progenitor cells are separated from mesenchymal stem/progenitor cells by a noticeably bright interstitial interface. The stem/progenitor cell niche as a whole is covered by few cell layers of the organ capsule. At the lateral side of the CD ampulla condensed mesenchymal cells are visible performing a mesenchymal-epithelial transition to develop into a renal vesicle, Comma- and then a S-shaped body as first visible signs of nephron development. Without exception all of demonstrated cells show the same light staining profile.At the neck of an ampulla the collecting duct tubule matures. At this site the primary development of well known light ‘Principal (P) Cells’ and somewhat darker labeled ‘Intercalated (IC) Cells’ can be seen (Figure 5c). As compared to light ‘Principal Cells’ neighboring ‘Intercalated Cells’ can be recognized by a slightly increased grey label of cytoplasm and by a barely increased amount of mitochondria. In so far ‘Intercalated Cells’ within the kidney do not appear to be identical with the dark cell type found in generated tubules (Figure 4c-f).

Further on, neonatal rabbit kidney fixed by glutaraldehyde including tannic acid was analyzed. Within the tip of a CD ampulla contained epithelial stem/progenitor cells are recognized. At the interstitial interface between the basal aspect of a CD ampulla and surrounding mesenchymal stem/progenitor cells intense label of tannic acid can be seen as it was described earlier (Figure 5b) [15]. Most important, in none of the cases label of tannic acid can be seen within the cytoplasm of cells. At the lateral side of a CD ampulla the label for tannic acid decreases so that the interstitial space at this site is free of label. Finally, at the neck of a collecting duct ampulla maturing ‘Principal Cells’ and ‘Intercalated Cells’ can be recognized. A slightly different grey label within the cytoplasm makes the difference (Figure 5d).Most important, after fixation in glutaraldehyde containing tannic acid the dark cells found in generated tubules could neither detected in the embryonic, maturing or matured parenchyma of the kidney. Further comparing earlier immunohistochemical profiles with tannic acid labeled cells in none of the cases a coincidence was found. Thus, neither in glomeruli, proximal, intermediate or distal portions of the nephron a comparable cell type was seen. In so far the dark cell type found in generated tubules has to be ascribed to abnormal development (Figure 4c-f).

## Discussion

Implantation of stem/progenitor cells into renal parenchyma appears as an attractive option to cure in future acute and chronic renal failure [20,21]. Although the concept sounds convincing, but a solid therapeutic basis is until now not in sight. Reasons for it are manifold comprising an up to date ineffective implantation technique, a suboptimal seeding and only a minimal survival of stem/progenitor cells in diseased renal parenchyma [22].

The injection of stem/progenitor cells into diseased parenchyma is the prerequisite to start the process of regeneration. However, the targeted application expects from stem/progenitor cells a complex adaption. During isolation their special niche environment is exchanged against a more or less suitable culture medium [5,23]. The situation becomes especially critical after infusion respectively injection. In contrast to the niche stem/progenitor cells are yet exposed to harmful interstitial fluid and altered extracellular matrix within diseased renal parenchyma [24-28]. In this unphysiological surrounding stimulating extracellular matrix and biochemical signals are missing sustaining normally stemness, proliferation, differentiation and development. Although all of these environmental conditions are poor, stem/progenitor cells have to seed and start with restoration. Thus, the challenge for the next future is consequently to elaborate an implantation technique that achieves on the one hand a protection against the unphysiological atmosphere in diseased parenchyma and supports on the other hand the initial seeding.

To gather basic data about environmental tolerance and individual physiological needs renal stem/progenitor cells were kept for the present investigation in advanced perfusion culture (Figure 1) [5]. For protection stem/progenitor cells were embedded in a fleece pad serving as an artificial interstitium [13]. Applying this concept the fibers of the polyester fleece mimic intact extracellular matrix, while the space between acts as a reservoir containing fresh interstitial fluid. Providing permanently fresh culture medium this technique produces a constant environment including a stable pH for a prolonged period of time. By varying experimental parameters developmental capacity of contained renal stem/progenitor cells can be tested. In a further step it is envisaged to implant the polyester pad under the renal organ capsule as a buffering reservoir for contained stem/progenitor cells.

In the actual series of experiments renal stem/progenitor cells were exposed to CO_2_ Independent Medium (Figure 2a,c) and Leibovitz’s L-15 Medium (Figure 2b,d). Although different in chemical composition, in both experimental series intense TROMA III label illustrates spatial development of numerous tubules within the artificial interstitium. Also semithin sections of specimens demonstrate occurrence of numerous tubules between fibers of the polyester fleece (Figure 3). Finally cross sections reveal that in regenerated tubules a lumen and a basal lamina are contained.Most interestingly, the kind of fixation displays quite new results. Specimens fixed by conventional glutaraldehyde exhibit an epithelium with a homogeneous cell population (Figure 3a,b). In contrast, fixation of specimens in glutaraldehyde containing tannic acid illustrates that tubules contain a heterogeneously composed epithelium consisting of bright and dark cells (Figure 3c,f). This important finding points out that conventional fixation by glutaraldehyde does not show all morphological details, while fixation by glutaraldehyde including tannic acid unmasks hidden morphological features.

Stem/progenitor cells cultured in CO_2_ Independent Medium (Figure 4a,c,e) and Leibovitz’s L-15 Medium (Figure 4b,d,f) show after fixation by glutaraldehyde that nucleus and cytoplasm of generated tubule cells appear normal. Intact mitochondria are orientated more to the basal than to the apical cell side of the cell. The apical and lateral plasma membranes were found in all cases to be separated by a tight junction complex. The lateral plasma membranes form narrow slits at the basal side speaking for an intact side to side contact of cells as it is found within the kidney. Further the basal plasma membrane of epithelial cells rests on a continuously developed basal lamina. In so far morphological analysis suggests that tubules generated in series with CO_2_ Independent Medium and Leibovitz’s L-15 Medium appear to be similar to renal tubule epithelium. In contrast, transmission electron microscopy displays also substantial inequalities. While after fixation with conventional glutaraldehyde a homogeneous tubule epithelium is seen consisting only of bright cells (Figure 4a,b), fixation by glutaraldehyde including tannic acid illustrates a heterogeneously composed epithelium consisting of bright and dark cells (Figure 4e-f). This observation was made as well in series with CO_2_ Independent Medium (Figure 4c,e) as in Leibovitz’s L-15 Medium (Figure 4d,f).

To obtain more information about identity, the dark cell type was searched in the rabbit kidney (Figure 5). Consequently, embryonic, maturing and matured parenchyma in neonatal kidney was investigated after fixation with conventional glutaraldehyde (Figure 5a,c) and by fixation with glutaraldehyde including tannic acid (Figure 5b,d). However, neither in the proximal tubule, the intermediate tubule, the distal, connecting or collecting duct tubule a dark cell type was registered. Only the known type of ‘Intercalated Cells’ in the connecting tubule and collecting duct epithelium was detected [29,30]. The primary development of an ‘Intercalated Cell’ can be seen in the neck of a ureteric bud derived collecting duct ampulla (Figure 5c,d). Thus, the dark cell type found in regenerated tubules fixed by glutaraldehyde containing tannic acid cannot be detected within the normal kidney.

A key question is whether illustrated darkly labeled tubule cells show features of normal development or whether up to date unknown pathological characteristics are contained. Formation of an excess of vacuoles as an indicator for cytotoxicity was not observed. In transmission electron microscopy it was further detected that the luminal cell border is clear so that the dark cells do not exhibit a luminal matrix. During tubulogenesis such a material is normally secreted by the surrounding epithelial cells to coordinate alterations in shape resulting in a correct lumen dimension [19]. Consequently, the lack of luminal matrix speaks for a differentiated cell. Moreover, in the illustrated dark cell type signs of programmed cell death (PCD) such as apoptosis or necroptosis cannot be recognized [31]. Finally, the intact appearance including integration within the epithelium indicates maintenance of differentiation. In so far the presently shown dark cell type found in generated tubules is unique and has to be ascribed to abnormal cell development.

## Conclusions

In the present experiments renal stem/progenitor cells were isolated to investigate regeneration of tubules under advanced in vitro conditions. The actual data exhibit that the kind of fixation for transmission electron microscopy displays quite new results. Specimens fixed by conventional glutaraldehyde exhibit an epithelium with a homogeneous cell population. In contrast, fixation of specimens in glutaraldehyde containing tannic acid illustrates that tubules contain a heterogeneously composed epithelium consisting of bright and dark cells. This important finding points out that conventional fixation by glutaraldehyde does not show all morphological details, while fixation by glutaraldehyde including tannic acid unmasks hidden morphological features.

## Competing interests

The authors declare no competing interests.

## Authors’ contributions

WWM coordinated the experiments, performed perfusion culture, analyzed specimens in fluorescence microscopy and interpreted results in transmission electron microscopy, designed the figure presentations and wrote the manuscript. LD isolated renal stem/progenitor cells, prepared media and the artificial interstitium, performed histochemical experiments, made special fixation, embedding, semi- and ultrathin sections and analysis in transmission electron microscopy. Further she made all works dealing with figure presentation. Both authors read and approved the final manuscript.

## Pre-publication history

The pre-publication history for this paper can be accessed here:

http://www.biomedcentral.com/1472-6890/14/34/prepub
